# Contribution of Imaging in Diagnosis of Primitive Cyst Hydatid in Unusual Localization: Pleura—A Report of Two Cases

**DOI:** 10.1155/2018/6242379

**Published:** 2018-07-09

**Authors:** Fatima Zahra Mrabet, Jihane Achrane, Yassir Sabri, Fatima Ezzahra El Hassani, Sanaa Hammi, Jamal Eddine Bourkadi

**Affiliations:** ^1^Department of Pneumology, Moulay Youssef University Hospital Center, Rabat, Morocco; ^2^Department of Parasitology, Ibn Sina University Hospital Center, Rabat, Morocco; ^3^Department of Cardiology, Military Hospital Mohamed V, Rabat, Morocco; ^4^Faculty of Medicine and Pharmacy, Mohamed V University, Rabat, Morocco

## Abstract

Hydatic disease has always been the most common in countries where large amount of sheep and cattle is raised, but increased travel and immigration have made this condition a serious worldwide public problem. Cyst hydatid may affect all parts of the human body like the heart, the bone marrow, the eye, the brain, the kidney, and the spermatic cord. Humans can become infested by accidentally ingesting the eggs that are passed in the feces from definitive hosts (usually a canid, such as a wolf, fox, or dog). Even in endemic countries, the primitive pleural hydatid cyst is exceptional, and it is very difficult to distinguish from other pleural and parietal cystic masses especially that in majority of cases the immunologic tests are negative. We report two cases of pleural hydatid cyst discovered in two young patients, with a nonspecific clinical presentation. The interest of this paper is to raise the primordial role of imaging in the positive diagnosis of primary pleural hydatid cyst.

## 1. Introduction

Hydatidosis is a parasitic disease caused by the development in humans of the larval form of Echinococcus Granulosus, a small dog tapeworm. Pleural localization is extremely rare even in endemic countries and represents only 1.3% of thoracic locations [1].

We report two observations of primary pleural hydatid cyst by insisting on the fundamental place of different imaging techniques in diagnosis.

## 2. Case Reports

### 2.1. Case Report (1)

**Figure 1:**
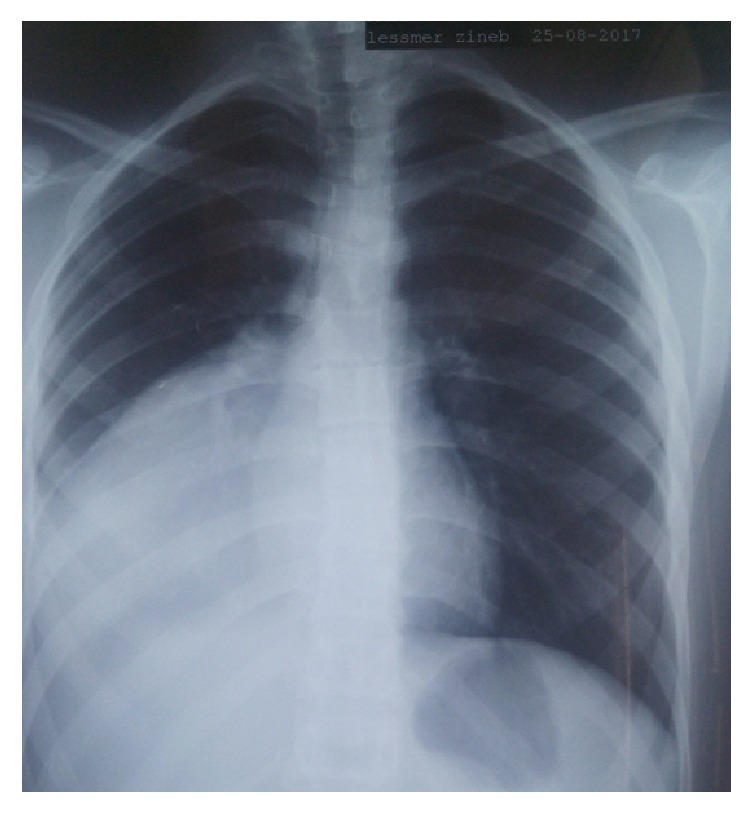
Posteroanterior chest roentgenogram showing a right basal opacity of watery tonality with convex upper limit (patient 1).

Miss A. L., a 17-year-old girl, with no pathological history and no notion of contact with dogs, reported since 3 months right thoracic pain, stage III of mMRC dyspnea, chest tightness, and some episodes of hemoptysis of low abundance evolving in a context of apyrexia, and conservation of the general state. The clinical examination revealed a right fluid effusion syndrome. The posteroanterior chest roentgenogram showed a homogeneous right basal opacity that effaced the diaphragmatic cupola and merged with mediastinum; its upper limit is convex (Figure 1).

**Figure 2:**
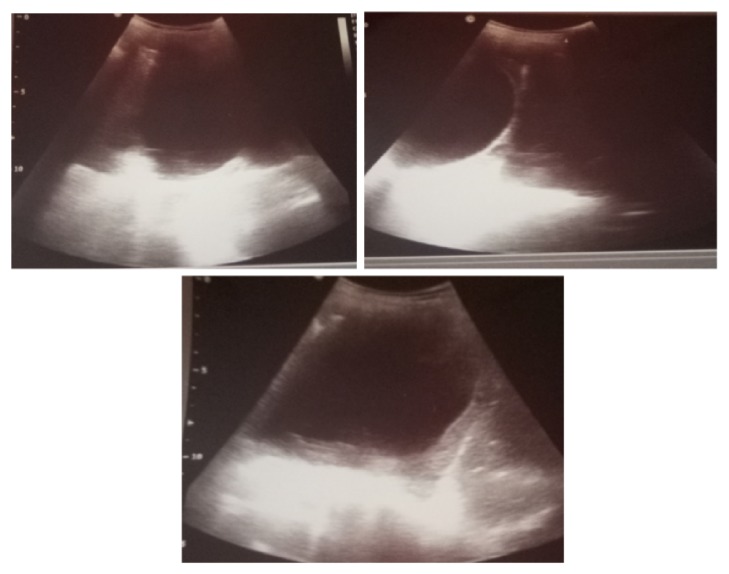
Thoracic ultrasound showing an intrapleural cyst with a proliferative membrane (patient 1).

Thoracic ultrasonography revealed an intrapleural cyst with a duplication of its wall suggesting a proliferative membrane without associated pleurisy (Figure 2).

**Figure 3:**
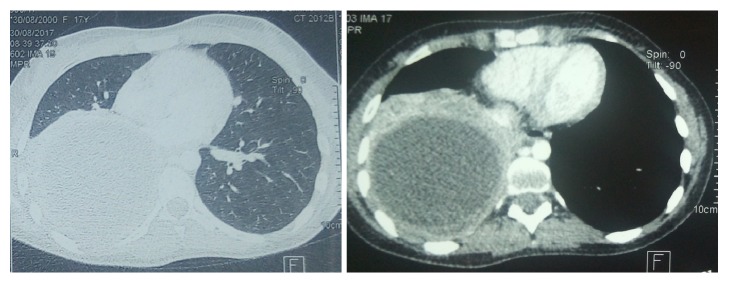
Thoracic CT scan showing a right basi-thoracic cystic image and a discreet slope with the pulmonary parenchyma with floating membrane aspect [right: parenchymal window, left: mediastinal window] (Patient 1).

Thoracic CT showed a right basal-thoracic cystic formation, measuring 126 *∗* 93 *∗* 93 mm, with a discreet slope with the adjacent parenchyma; its wall was thickened and enhanced after injection of contrast product. The lung parenchyma was without anomaly with the exception of passive atelectasis adjacent to the cyst, confirming the diagnosis of a right pleural cyst type II of Gharbi classification (Figure 3).

**Figure 4:**
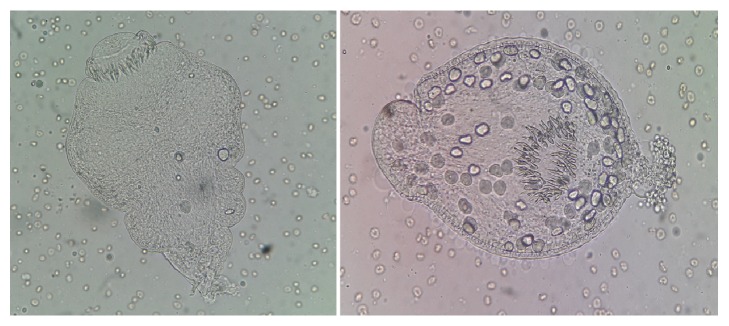
Parasitological study of the cyst fluid showing scolex [right: evaginated scolex, left: invaginated scolex] (Patient1).

The blood count was normal and the ELISA and Indirect Agglutination serologies were negative. In a second stage, the research for other localizations of the hydatid cyst was negative (abdominal ultrasound, echocardiography, and cerebral CT), hence the primitive character of pleural hydatidosis in our observation. During surgery, the presence of a cystic formation in the parietal pleural was noted. The delicate dissection had objectified thickened visceral pleura. The cystectomy was successfully performed without rupture and the piece was sent to the parasitology laboratory with evidence of proliferative membrane (macroscopically) and alive scolex in the intracystic fluid (microscopically) (Figure 4).

### 2.2. Case Report (2)

**Figure 5:**
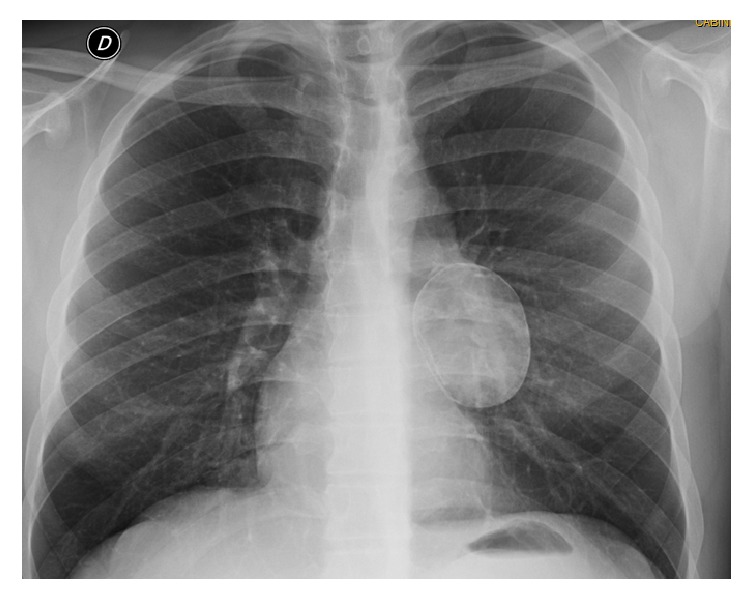
Posteroanterior chest roentgenogram elucidating a homogeneous oval opacity of water tonality, well limited, left hilar (Patient 2).

Mr. SF, a 26-year-old man, without any notable pathological history, have a notion of contact with dogs in childhood, asymptomatic on the respiratory plane. The posteroanterior chest roentgenogram was performed for him as a preemployment checkup. It objectified a homogeneous oval opacity, well limited, left hilar, and having the internal edge in intimate contact with the left edge of the heart (Figure 5).

**Figure 6:**
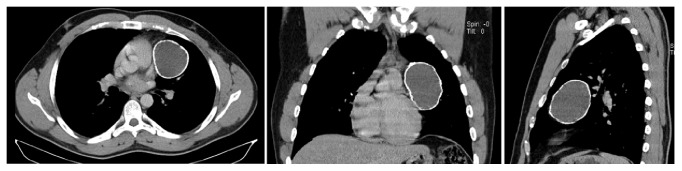
Thoracic CT showing left anterolateral mediastinal mass with total parietal calcification (mediastinal window) [right: horizontal section, middle: frontal section, and left: sagittal section] (Patient2).

In this context, a chest CT scan revealed a left anterolateral mediastinal mass with a total parietal calcification measuring 70 mm in height and 55 mm in lateral diameter (Figure 6).

**Figure 7:**
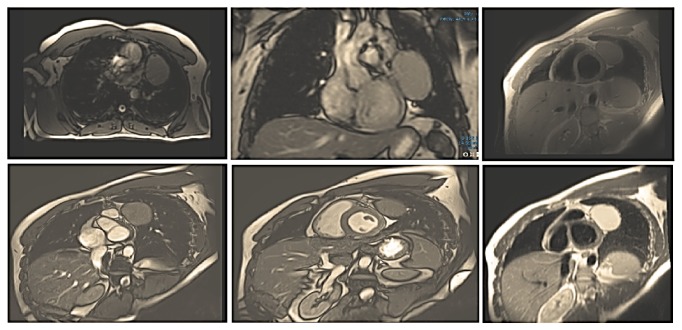
Cardiac MRI showing a para cardiac cyst next to the anterolateral wall of the left ventricle in close contact with the pericardium but with a cleavage plane (Patient 2).

Echocardiography confirmed the presence of left-ventricular extracardiac structure without intracavitary lesion or associated pericardial effusion. Likewise, magnetic resonance imaging (MRI) showed a mediastinal cyst next to the anterolateral wall of the left cardiac ventricle, in close contact with the pericardium but with a cleavage plane and no mass effect on the cardiac cavities, measuring 72mm *∗* 53mm. Its tonality was hypointense on T1 and hyperintense on T2 (Figure 7).

The blood count was normal and the ELISA and Indirect Agglutination serologies were negative. In a second stage, the research for other localizations of the hydatid cyst was negative (abdominal ultrasound and cerebral CT), hence the primitive character of pleural hydatidosis in this second observation. In operation, the heart was of normal volume with no intrapericardial mass. At the opening of the left pleura, the exploration found a solid mass contiguous to the mediastinal pleura and in contact with the left phrenic nerve. The careful dissection and excision of the mass were successfully performed without complications.

## 3. Discussion

The hydatid cyst is a parasitic disease that is still endemic in several parts of the world, especially around the Mediterranean rim. The lung constitutes the second hydatid localization (20 to 40%) after the liver (75%). The primary pleural localization is exceptional, representing only 1.3% of thoracic locations [2]. It mostly affects the young adult male. We reported two observations of two young patients of different sex.

Once a human has been infested with the taenia eggs, gastric and enteric digestion facilitates the release of larvae, which penetrate the intestinal wall until they reach a small vessel system. Passing through the bloodstream, they arrive at the organ where they can settle and transform into small cysts that increase in size by 2 to 3 cm per year. The usual locations are the liver and lungs; intrathoracic but extrapulmonary locations like the pleura, diaphragm, mediastinum, pericardium, and chest wall are uncommon. Pleural hydatid cysts can develop chiefly as a result of liver or lung cyst rupture into the pleural space with complications of pneumothorax, pleural effusion, or empyema [3]. With taking respiration, eggs settle in the lungs distally. In the humid environment, they become scolexes and pass the alveolocapillary membrane and join systemic circulation by pulmonary veins, form primary isolated cysts in organs such as heart, bone marrow, eye, and brain. But some of scolexes may move into the pleural space with negative pleural pressure and settle there, causing disease. It is probably the case in the patients presented. In the pleura, cysts sit between the parietal pleura and the endothoracic fascia, and the involvement appears to be systemic or lymphatic [4]. The pleural layers are avascular, and a hydatid cyst may form and grow in this region because the structure of the laminated cyst membrane is permeable to calcium, potassium, chloride, water, and urea. Accordingly, these nutritional substances and others that may be useful to the parasite can traverse the membrane via diffusion. Active transport may be involved in this process [5, 6].

The clinical symptomatology is poor and nonspecific; it can simulate any pleural-pulmonary disease (chest pain, dyspnea, and dry cough). The diagnosis can be made in the acute phase in front of a symptomatology of sudden onset of thoracic pain and dyspnea, following the rupture of the cyst. In other cases, this new location may remain asymptomatic for a long time and the diagnosis can be done tardily. Exceptionally, there may exist some signs of mediastinal compression depending on the location. The discovery can also be fortuitous on a chest roentgenogram [1, 7]. As reported in the literature, our first patient had a discreet clinical presentation, while the second patient was outright asymptomatic.

Imaging is a fundamental element for positive diagnosis. The chest roentgenogram shows a homogeneous pleural opacity, well-defined with water tonality. Rarely, it shows peripheral calcifications that it orientates the diagnosis. In some cases, radiography may guide the diagnosis at an early stage by showing characteristic iconography, such as the presence of cystic formation having a calcified wall, which is rare but represents a strong diagnostic presumption; the chest roentgenogram in our second patient was very characteristic and diagnosis was very probable; the other investigations had an objective to determine the exact localization of the cyst (cardiac or pleural). Ultrasound is a second-line examination; it is very efficient with a diagnostic specificity estimated at 96%. It can make the diagnosis and make the extension assessment and the surveillance, as the case of our first patient [1]; it can show multicystic or hydroaerial images when the hydatid cyst is broken. In many cases, it shows the proliferative membrane doubling inside the pericyst which is pathognomonic of hydatid cyst, confirms the liquid nature of pleural opacity, and evokes positive diagnosis especially in case of multivesicular form; it also makes it possible to detect a possible pleural effusion associated with it and to look for other cystic localizations, particularly abdominal ones. Computed tomography confirms the pleural localization of the well limited fluid mass unmodified by injection of the contrast product; it is more sensitive than the previous modalities and makes it possible to pose the diagnosis with a higher specificity and sensitivity [1]. MRI provides diagnostic support in cases where cysts are not characteristic on ultrasound or CT, especially in pseudo-tumoral forms [1]. MRI makes it possible to better define the topography of the cyst and its relations with the neighboring organs thanks to the multiplanar sections. The T2-weighted sequences visualize small daughter vesicles or a floating membrane in favor of the diagnosis and especially a peripheral T1 and T2 hyposignal in relation to the calcium deposits which is pathognomonic to the diagnosis [2].

Biologically, hypereosinophilia is generally absent in cases of intrathoracic hydatid disease. Immunological tests such as IgG ELISA, indirect hemagglutination, and Western Blot may be helpful, but their sensitivity is only about 60%. A combination of two or more biological tests and radiological imaging should be used for accurate diagnosis [6]. The sensitivity of the immunology increases significantly in case of complication or associated liver cyst. Our two patients had negative immunologic tests similar to that described in the literature.

In the absence of rupture, the puncture of the cyst is formally contraindicated, which explains why cytological or pathological diagnosis is usually performed only after its surgical excision [8].

The positive diagnosis is difficult to establish due to the rarity of the condition and the lack of clinical, radiological, and biological specificity. Indeed, several other causes of cystic lesions can be evoked: bronchogenic cyst, enteric cyst, pleuropericardial cyst, thymic cyst, and lymphangioma. The diagnosis of certitude is almost always operative by visualizing the hydatid membrane and/or daughter's vesicles or after pathological study of the operative piece in case of infected or thickened cyst [3]. The mobilization of the daughter vesicles has been considered as a sign of secondary pleural hydatidosis and, to our knowledge, such mobilization has not been observed in the primitive forms [9]. In our two observations, imaging played a fundamental role and the positive diagnosis was almost certain even preoperatively; otherwise, the surgery and the parasitological study of the operative piece came to set the diagnosis and validate the data already obtained by imaging.

As a general rule, when the presurgical diagnosis of hydatid cyst is suspected, surgeon should take care of four things in order to achieve complete resection and to avoid recurrence of disease from pleural hydatid cysts:

(i) Three days before emergency resection if necessary and 1 week before elective surgery albendazole treatment should start to increase the blood and tissue concentration of the medicine in case of the risk of contamination

(ii) Plan the appropriate surgical approach to prevent cystic rupture or spillage when doing the thoracotomy.

(iii) Inactivate daughter cysts and scolices prior to removal by injecting 20% hypertonic saline solutions into the cyst.

(iv) To give the anticoloidal agent into the cyst, it is necessary to empty some of the cyst content; otherwise, it will leak out of the injection site during surgery. Distention may be reduced by aspiration, and this will ease the manipulation and surgical dissection.

(v) Do not spill cyst contents during surgery to avoid anaphylactoid reaction, recurrence, and multiple hydatidosis.

(vi) Completely remove of the cyst including the innermost germinative layer, which can produce scolices, with en-bloc excision whenever possible; sometimes, to avoid recurrence, it is necessary to resect the affected surrounding tissues completely.

Postoperatively, it may be necessary to place patients on an anthelmintic medical regimen (Albendazol) with appropriate follow-up reevaluations [10].

## 4. Conclusion

The primary pleural localization of the hydatid cyst is very rare or even exceptional. Its discovery is often fortuitous. More rarely, primary pleural hydatid cyst is symptomatic with a discreet and nonspecific clinical presentation. Imaging especially in section, plays a fundamental role; it makes it possible to guide the diagnosis, to specify the topography and the relations with the neighboring organs, and to look for other localizations.

The feature of our observations, compared to literature, is the similarity to the young age, the discreet clinical presentation, the absence of hypereosinophilia, the negative hydatid serology, and especially the diagnostic orientation thanks to imaging.

## Data Availability

The author agrees to share all the data

## Abbreviations

mMRC: Modified Medical Research Council
CT: Computed Tomography
ELISA: Enzyme-Linked Immunosorbent Assay
MRI: Magnetic Resonance Imaging
Ig G: Immunoglobulin G.
